# Supplementary material for: Non-surgical treatments for post-burn scars: A network meta-analysis
Source: PLoS One. 2025 Aug 21;20(8):e0330428. doi: 10.1371/journal.pone.0330428 (PMC12370048; doi:10.1371/journal.pone.0330428)
Supplement: S2 File — (DOCX) [file pone.0330428.s002.docx]

**S2 file. Search strategy**

Search strategy in PubMed

| Steps | Search strategy |
| --- | --- |
| #1 | (((burns[MeSH Terms])) OR (burn[Title/Abstract])) OR (thermal injury [Title/Abstract]) |
| #2 | ((((cicatrix [MeSH Terms])) OR (cicatrization [Title/Abstract])) OR (scars [Title/Abstract])) OR (scar* [Title/Abstract]) |
| #3 | #1 AND #2 |
| #4 | ((((((random allocation [MeSH Terms])) OR ( randomized controlled trial [Title/Abstract])) OR ( randomised controlled trial[Title/Abstract])) OR (random*[Title/Abstract])) OR (randomized[Title/Abstract])) OR (randomised [Title/Abstract]) |
| #5 | #3 AND #4 |

Search strategy in Web of Science

| Steps | Search strategy |
| --- | --- |
| #1 | ((TS=(burns)) OR TS=(burn)) OR TS=(thermal injury) |
| #2 | (((TS=(cicatrix)) OR TS=(cicatrization)) OR TS=(scars)) OR TS=(scar*) |
| #3 | #1 AND #2 |
| #4 | (((((AB=(random allocation)) OR AB=(randomized controlled trial)) OR AB=(randomised controlled trial)) OR AB=(random*)) OR AB=(randomized)) OR AB=(randomised) |
| #5 | #3 AND #4 |

Search strategy in Embase

| Steps | Search strategy |
| --- | --- |
| #1 | 'burn'/exp |
| #2 | 'burns':ab,ti OR 'thermal injury':ab,ti |
| #3 | #1 OR #2 |
| #4 | 'scar'/exp |
| #5 | 'cicatrix':ab,ti OR 'cicatrization':ab,ti OR 'scars':ab,ti |
| #6 | #4 OR #5 |
| #7 | #3 AND #6 |
| #8 | 'randomization'/exp |
| #9 | 'randomized controlled trial':ab,ti OR 'randomised controlled trial':ab,ti OR 'random':ab,ti OR 'randomized':ab,ti OR 'randomised':ab,ti OR 'random allocation' |
| #10 | #8 OR #9 |
| #11 | #7 AND #10 |

Search strategy in Cochrane Library

| Steps | Search strategy |
| --- | --- |
| #1 | "burns":ti,ab,kw OR "burn":ti,ab,kw OR "thermal injury":ti,ab,kw |
| #2 | "scar":ti,ab,kw OR "scars":ti,ab,kw OR "cicatrix":ti,ab,kw OR "cicatrization":ti,ab,kw |
| #3 | #1 AND #2 |
| #4 | "random allocation":ti,ab,kw　OR　" randomized controlled trial":ti,ab,kw OR "random":ti,ab,kw OR "randomized":ti,ab,kw |
| #5 | 3# AND #4 |

Search strategy in PEDro

| Steps | Search strategy | |
| --- | --- | --- |
| #1 | Abstract & Title | scars |
| #2 | Problem | skin lesion, wound, burn |
| #3 | Method | clinical trial |
| #4 | When Searching | 1# AND #2 AND #3 |
