# Supplementary material for: Non-surgical treatments for post-burn scars: A network meta-analysis
Source: PLoS One. 2025 Aug 21;20(8):e0330428. doi: 10.1371/journal.pone.0330428 (PMC12370048; doi:10.1371/journal.pone.0330428)
Supplement: S3 File — (DOCX) [file pone.0330428.s003.docx]

**S3 File. Data.**

**Analysis data on the improvement of VSS score by non-surgical treatment.**

| **id** | **study** | **t** | **mean** | **sd** | **n** |
| --- | --- | --- | --- | --- | --- |
| 1 | Roh et al (2007) | MT | -4.71 | 3.12 | 18 |
|  |  | SMC | 0.35 | 1.8 | 17 |
| 2 | Harte et al (2009) | PTS | 3.181 | 2.750 | 10 |
|  |  | PT | 2.454 | 2.252 | 9 |
| 3 | Li-Tsang et al (2010) | PT | -1.4 | 2.10 | 36 |
|  |  | SMC | 0.5 | 2.08 | 9 |
| 4 | Steinstraesser et al (2011) | PTS | -4.1 | 1.61 | 19 |
|  |  | PT | -3.7 | 1.87 | 19 |
| 5 | Blome-Eberwein et al (2014) | CO2 LT | -0.96 | 2.52 | 36 |
|  |  | SMC | -0.47 | 2.56 | 36 |
| 6 | Zaghloul et al (2016) | ESWT+SMC | -4.25 | 1.72 | 20 |
|  |  | SMC | -1.25 | 1.70 | 20 |
| 7 | Ouyang et al (2018) | CO2 LT+PDL | -6.75 | 1.28 | 28 |
|  |  | PDL | -5.83 | 1.30 | 28 |
| 8 | Elrashid et al (2018) | OPLT+SMC | -3.99 | 2.15 | 15 |
|  |  | SMC | -1.86 | 1.84 | 15 |
| 9 | Mehran et al (2019) | Microneedling | -2.83 | 2.13 | 30 |
|  |  | CO2 LT | -1.4 | 2.07 | 30 |
| 10 | Joo et al (2020) | ESWT+SMC | -0.35 | 1.61 | 23 |
|  |  | SMC | 0.32 | 1.75 | 25 |
| 11 | Kivi et al (2024) | CO2 LT+PDL | 0 | 8.78 | 25 |
|  |  | CO2 LT | 0 | 8.15 | 19 |
|  |  | PDL | -0.24 | 8.18 | 18 |

**Analysis data on the improvement of VAS score by non-surgical treatment.**

| **id** | **study** | **treatment** | **mean** | **sd** | **n** |
| --- | --- | --- | --- | --- | --- |
| 1 | Li-Tsang et al（2010） | PTS+SMC | -1.42 | 2.03 | 24 |
|  |  | PT+SMC | 0.42 | 2.85 | 26 |
|  |  | SG+SMC | -0.77 | 2.02 | 22 |
|  |  | SMC | 0.12 | 2.35 | 12 |
| 2 | Parlak et al（2010） | MT+SMC | 3.59 | 1.26 | 32 |
|  |  | SMC | 0.12 | 0.34 | 31 |
| 3 | Cho et al（2014） | MT+SMC | -2.61 | 1.28 | 76 |
|  |  | SMC | -1.18 | 1.42 | 70 |
| 4 | Ebid et al（2017） | HILT | -4.11 | 3.69 | 24 |
|  |  | SMC | -0.78 | 3.55 | 25 |
| 5 | Joo et al（2020） | ESWT+SMC | -1.48 | 1.04 | 23 |
|  |  | SMC | -0.52 | 0.77 | 25 |

**Analysis data on the improvement of scar thickness by non-surgical treatment.**

| **id** | **study** | **t** | **mean** | **sd** | **n** |
| --- | --- | --- | --- | --- | --- |
| 1 | Li-Tsang et al（2010） | PTS | -1.76 | 2 | 24 |
|  |  | PT | -0.92 | 2.43 | 26 |
|  |  | SG | -1.15 | 1.45 | 22 |
|  |  | SMC | -0.76 | 1.73 | 12 |
| 2 | Blome-Eberwein et al（2016） | CO2 LT | -0.81 | 0.35 | 36 |
|  |  | SMC | -0.198 | 0.343 | 36 |
| 3 | Zaghloul et al（2016） | ESWT+SMC | -2.86 | 1.41 | 20 |
|  |  | SMC | -0.8 | 1.32 | 20 |
| 4 | Nedelec et al（2019） | MT+SMC | -0.31 | 0.93 | 60 |
|  |  | SMC | -0.221 | 1.837 | 60 |
| 5 | Joo et al（2020） | ESWT+SMC | 0.01 | 0.07 | 23 |
|  |  | SMC | 0.07 | 0.07 | 25 |
| 6 | Wiseman et al（2020） | PTS | 0.06 | 0.12 | 48 |
|  |  | SG | 0.01 | 0.09 | 51 |
|  |  | PT | 0.02 | 0.09 | 43 |
| 7 | Lee et al（2021） | ESWT+SMC | 0 | 0.01 | 25 |
|  |  | SMC | 0.06 | 0.09 | 23 |
