# Supplementary material for: Non-surgical treatments for post-burn scars: A network meta-analysis
Source: PLoS One. 2025 Aug 21;20(8):e0330428. doi: 10.1371/journal.pone.0330428 (PMC12370048; doi:10.1371/journal.pone.0330428)
Supplement: Attachment — Submitted filename: Response to Reviewers.docx [file pone.0330428.s005.docx]

Dear Editors and Reviewers,

Thank you very much for giving us opportunities to revise our manuscript, and we appreciate the reviewer a lot for his positive and constructive comments and suggestions. We have studied reviewer’s comments carefully and have made revisions. We hope the corrections will meet with your approval.

Reviewer 1:

***Comment 1: The paper includes impressive statical analysis and offers new evidence that would add towards burn scar care. However, I feel this paper is written poorly and needs thorough revisions to improve its overall message and strength its evidence.***

**Response**: Thank you very much for your comprehensive review and constructive feedback on our manuscript. We are delighted to learn that you find our statistical analysis commendable and that our new evidence makes a valuable contribution to the field of burn scar care.

We acknowledge your concerns regarding the writing quality of the paper, as well as the necessity for thorough revisions to enhance its overall message and strengthen the presented evidence. We take your comments seriously and appreciate your guidance in assisting us with improving the manuscript.

In response to your feedback, we have refined the language to ensure clarity, precision, and coherence. Additionally, we have worked on tightening the structure and flow of the paper, ensuring that each section effectively contributes to conveying our overall message. Furthermore, we have meticulously reviewed all evidence presented in the manuscript to guarantee that every claim is substantiated by data and analysis.

***Comment 2: The paper feels informal in nature, and I would advise the authors to remove ‘we’ in the context of the authors decision throughout the paper. Please note not to start sentences with a number. Furthermore, I feel the authors writing style could be more concise to allow for more characters to defend and boarded their discussion points.***

**Response**: Thank you very much for your detailed feedback and constructive suggestions. We have removed the use of the pronoun “we” throughout the manuscript to maintain a more formal and objective tone. This change ensures that the manuscript adheres to the standard academic writing conventions and focuses on the presentation of the research findings rather than the authors’ personal perspective. And we have revised the manuscript to avoid starting sentences with numbers. We have rephrased such sentences to ensure they comply with proper grammatical conventions while maintaining clarity and readability. In addition, we have streamlined the text to eliminate unnecessary wording and redundancy, allowing us to allocate more space for a more in-depth discussion of our findings. This includes a more detailed analysis of the implications of our results and a broader exploration of the relevant literature to support our conclusions.

Thank you once again for your valuable feedback, which has helped us refine the manuscript.

***Comment 3: ‘hyperplastic scar’ is the umbrella terminology for keloid, hypertrophic and cheloid scars. When the authors refer to keloid and hyperplastic scars in the text they should discuss as just hyperplastic scarring or keloid and hypertrophic scars.* Response**: We sincerely appreciate the reviewer’s insightful feedback and meticulous identification of the terminological imprecision regarding scar classifications. In light of this comment, we review literature and recognize that the concomitant use of “keloid” and “hyperplastic scars” may introduce ambiguity, as the latter serves as an overarching category that includes keloid, hypertrophic, and cheloid scars. We fully concur with the reviewer’s recommendation. To rectify this inconsistency and enhance terminological rigor, we will use “keloids and hypertrophic scars” instead of “keloid and hyperplastic scars” in this study.

***Comment 4: Finally, despite referencing Table 1 (characteristics) and 3 – no tables have been attached to the submission. These would have to be reviewed before final acceptance. I would also hope a table offering a brief overview of each paper would add to the understanding of the paper.* Response**: Thank you very much for bringing this to our attention and for your constructive suggestion regarding the missing tables. We deeply apologize for the oversight in not attaching Tables 1 and 3 to our submission. We understand the importance of these tables, and we will immediately rectify this by attaching the missing tables to the submission.

Furthermore, we appreciate the reviewer's thoughtful suggestion to include a table summarizing each referenced paper, as such a framework could indeed enhance readability in certain scenarios. However, given the journal's rigorous words constraints and our intentional effort to streamline the narrative, we have strategically integrated key details of each study into Table 1, aiming to balance comprehensiveness with the publication's formatting guidelines. Should the editorial team believe a dedicated overview table would better align with the manuscript's objectives, we would gladly revisit this decision during revisions.

***Comment 5: Abstract***

***Background and aim: ‘This study aims to compare the outcomes of non-surgical treatmentS for post-burn scars using network meta-analysis.’ This will correlate to your title.*** Response: Thanks for your comment. We understand the importance of maintaining consistency across the manuscript. We make the necessary revisions to ensure that the title, background, and objectives clearly reflect the core focus of our research. The revised “Background and aim” as shown below:

Post-burn scarring is a prevalent condition, and the existing non-surgical treatments exhibit varying degrees of efficacy. There is limited evidence available to determine the effective non-surgical treatment for post-burn scars. This study employs a multi-index network meta-analysis to conduct a comprehensive evaluation and comparative ranking of non-surgical treatments for post-burn scars. The aim is to identify the most effective treatment methods, thereby providing a robust, evidence-based foundation to guide clinical decision-making.

***Comment 6: Introduction***
***I feel the first paragraph should be re-written to deliver a more gripping and comprehensive overview of burn scar care.***

**Response**: Thanks for your valuable feedback on improving the clarity and impact of the introduction. We fully agree that a compelling opening paragraph is critical to contextualizing the importance of burn scar care. In response to your suggestion, we have revised the first paragraph to:

Burn injuries present a significant global healthcare challenge, with 30% to 90% of survivors developing pathological scarring, predominantly in the form of keloids and hypertrophic scars. These conditions disrupt the intricate process of physiological wound repair [1]. Such aberrant healing responses not only lead to chronic pain, persistent itching, and joint contractures but also severely impact psychosocial well-being and functional capacity, thereby significantly reducing quality of life [2]. Effective scar management emerges as a critical determinant of recovery outcomes in burn patients [3], particularly within the first year after injury, when scars are immature and more responsive to treatment. Currently, non-surgical treatments are often the initial treatment of choice for inhibiting or slowing scar progression [4-6].

***Comment 7: More explanation concerning the benefits of non-surgical technique should be explored to highlight the importance of what your evidence will influence in burn care.***

**Response**: Thanks for your valuable suggestion regarding the need for a more detailed exploration of the benefits of non-surgical techniques. We strongly agree that highlighting these advantages strengthens the rationale for prioritizing evidence-based non-surgical strategies. We have expanded our explanation to emphasize key benefits, including: risk reduction, versatility, accessibility, psychosocial benefits and cost-effectiveness, etc. These changes are on pages 3-4 of the revised manuscript, as shown below:

Silicone products, pressure therapy (PT), massage therapy (MT), and extracorporeal shock wave therapy (ESWT) are widely recognized non-surgical treatments for post-burn scars [7]. These interventions have demonstrated significant efficacy in improving both the symptoms and appearance of post-burn scars, while also being well-tolerated by patients [8-10]. In addition to the aforementioned non-surgical treatments, CO2 laser therapy (CO2LT) and microneedling can be considered important components of non-surgical treatment strategies for post-burn scars [11]. CO2LT is used to resurface the skin, promoting collagen remodeling and improving scar texture and appearance. Though it involves a higher degree of invasiveness compared to silicone products or PT, it does not typically require general anesthesia, especially when applied in fractional modes. Microneedling involves creating micro-injuries to stimulate collagen production and improve scar healing. Although it requires penetration of the skin, microneedling can be performed under local anesthesia. Despite their relatively higher invasiveness levels compared to other modalities mentioned earlier, CO2LT and microneedling do not involve surgical tissue removal, thus they remain classified as non-surgical treatments.

Non-surgical techniques offer several key advantages that make them highly relevant in modern burn care. Firstly, these methods are minimally invasive or non-invasive, which reduces the risk of complications and enhances patient safety. Secondly, compared to surgical options, they are often easier to implement, making them accessible in various clinical settings. Thirdly, these treatments can significantly improve patients’ quality of life by alleviating pain, reducing scar stiffness, and enhancing cosmetic outcomes. By focusing on non-surgical interventions, healthcare providers can offer effective solutions that align with patient preferences for less invasive treatments, while also minimizing recovery time and improving resource utilization. The growing adoption of these therapies underscores their potential to influence comprehensive, patient-centered burn care strategies [12].

***Comment 8: Many sweeping statements are made addressing the reliability and validity of VSS and VAS. These tools are widely debated as to their reliability and validity and this is the main reason centres now choose the POSAS score to objectively measure scar care. The authors need to add more evidence to support their conclusion concerning VSS and VAS.***

**Response**: Thank you for your thoughtful feedback. We fully acknowledge the ongoing debate about the reliability and validity of these tools, particularly in comparison to more comprehensive instruments like POSAS. In designing this network meta-analysis, our selection of VSS and VAS as primary outcomes was driven by their widespread use in existing randomized controlled trials (RCTs) on non-surgical burn scar treatments. However, we agree that these scales have inherent limitations, including inter-rater variability and subjectivity (particularly for VAS). We have revised the manuscript to address your concerns as follows:

Evaluating the efficacy of treatments for post-burn scars is crucial for guiding clinical decision-making and enhancing therapeutic outcomes. To achieve this, various assessment tools have been developed to evaluate different aspects of scar characteristics. The Vancouver Scar Scale (VSS) is one of the most commonly used tools for assessing scar characteristics, including vascularity, pliability, pigmentation, and height [13]. Although VSS has undergone extensive review and remains widely utilized, available data on its reliability have received indeterminate quality ratings [14]. Furthermore, VSS may lack sensitivity to subtle changes in scar appearance, and its subjective components can lead to variability in scoring [15]. Despite these limitations, studies have shown that VSS demonstrates moderate inter-rater reliability when applied by trained evaluators [16]. The Visual Analog Scale (VAS) is another essential tool, primarily used for assessing pain and discomfort associated with scars. It quantifies a patient’s subjective experience by asking them to indicate their pain level on a linear scale. The simplicity and ease of use of VAS make it a popular choice for pain assessment [17]. However, VAS scores are heavily reliant on patient self-reporting, which can be influenced by individual biases or external factors. Scar thickness serves as an objective indicator that can be accurately measured using ultrasound or other imaging techniques [18]. Monitoring scar thickness enables clinicians to adjust treatmsent regimens, such as increasing the frequency of interventions or modifying treatment methods, thereby optimizing therapeutic effectiveness. Collectively, the VSS captures morphological and physiological scar characteristics, the VAS quantifies patient-reported symptomatic experiences, and scar thickness measurements offer objective structural insights. These metrics address the multidimensional nature of post-burn scars evaluation, enabling comprehensive analysis of non-surgical treatment impacts.

***Comment 9: Again, a statement has been made concerning previous studies have ‘some limitations’ with no supporting discussion. If the authors want to improve from previous literature, they must first comment and address previous literature limitations thoroughly.***

**Response**: We appreciate your feedback regarding the need to thoroughly address the limitations of previous studies rather than simply stating that they had “some limitations”. To better reflect this guidance and enhance our manuscript, we will revise the relevant section to provide a more detailed discussion of these limitations. These changes are on page 6 of the revised manuscript, as shown below:

Although previous systematic reviews have provided valuable insights into non-surgical treatments for post-burn scars, a clear understanding of their specific effectiveness remains elusive. Several key limitations in prior studies contribute to this gap. First, many studies utilized different methodologies, outcome measures, and follow-up durations, complicating efforts to draw definitive conclusions about the comparative efficacy of various treatments. Second, several earlier studies were limited by small sample sizes, which reduced their statistical power and generalizability. Finally, the inconsistent use of assessment tools across studies introduced variability in how outcomes were measured and reported, further hindering meaningful comparisons.

***Comment 10: Overall, I feel the introductions is still in draft form and should be strengthen to provide a clearer picture of current burn scar care.***
 **Response**: Thanks for your valuable comments. We agree that the introduction can be further strengthened to provide a clearer picture of current burn scar care. We revise this section to include more detailed information on existing treatment modalities, their limitations, and the gaps in current research to better contextualize our study.

***Comment 11: Method***
***Please could the authors reflect on why they did not include ‘keloid’ and ‘hypertrophic’ within their key word search despite this being the topic of the paper. It may be sensible to re-run a search and ensure no texts have been missed.***
 **Response**: Thanks for your comments. Tough we appreciate the suggestion, we would like to clarify the rationale for not explicitly including “keloid” and “hypertrophic” as standalone keywords:

(1) Despite not using “keloid” or “hypertrophic” as keywords, our search strategy was designed to be highly sensitive. By combining burn-specific terms with “cicatrix”, “cicatrization”, and “scars”, we captured a broad spectrum of post-burn scar literature. Additionally, manual screening of reference lists and relevant systematic reviews further mitigated the risk of missing eligible studies.
(2) Had we included “keloid” or “hypertrophic” as keywords, we risked retrieving a large volume of irrelevant studies, which would have increased screening burden and potentially diluted the focus on non-surgical burn scar management.
(3) We believe that our current search strategy adequately captured the literature relevant to our research question. However, to address your concern transparently, we have re-examined the retrieved studies and confirmed that no eligible texts were missed due to the absence of these terms.

***Comment 12: Did the author attempt to contact the papers’ authors if full text was unavailable? If no, why not? If yes, please state in the manuscript.***
 **Response**: Thank you for highlighting this important point. In our study, we successfully retrieved all identified articles in full text through comprehensive access provided by institutional subscriptions and open-access platforms such as PubMed, Web of Science, Cochrane Library, and Embase. Throughout the search and screening process, we did not encounter any instances of unavailable full texts.

***Comment 13: Please clarify why the authors choose to extract data at the 6-month point. What statistical guidance in burn care pointed to this being the optimum treatment efficacy***?
 **Response**: Thank you for your insightful comment. Upon re-examining the studies included in our analysis, we found that the majority of studies focused on comparing outcomes before and after the intervention, rather than reporting data at multiple timepoints. Only a small subset of studies provided longitudinal data across several timepoints. As a result, extracting data consistently across all studies was challenging. To ensure a robust and standardized comparison, we chose to focus on a single timepoint (closer to 6 months post-burn), which is widely regarded as a clinically relevant period for assessing scar outcomes during the remodeling phase. We fully acknowledge the limitation of using a single timepoint, as it may not reflect the dynamic process of scar maturation or the potential impact of interventions on the trajectory of improvement. In light of your feedback, we have added a discussion point in the revised manuscript addressing this limitation, as shown below:

We now explicitly note that the use of a single timepoint may not capture the full temporal effects of interventions such as PT or MT on scar maturation and symptom improvement. We emphasize the need for future studies to adopt standardized longitudinal reporting across multiple timepoints (such as 3 months, 6 months, and 12 months post-burn) to better assess the progression of scar outcomes and the sustained effects of interventions.

***Comment 14: Correct the references for software and Cochrane Manual to be included in the reference list.***
 **Response**: Thanks for your comments. According to your suggestions, we have reviewed all software references cited in our manuscript and ensured that they are formatted according to the appropriate citation style. We have also verified that the versions of the software mentioned are accurately cited. The corrected references have been included in the updated reference list. In addition, We have located the specific edition of the Cochrane Manual that was referenced in our work and have ensured that it is cited correctly in the text and included in the reference list. These changes are shown below:

(1) Page 9: Two independent reviewers (XRM and XTX) assessed the risk of bias for all included studies using the Cochrane Risk of Bias Tool [24].
(2) Page 9: A frequentist NMA was conducted in this study by use of Stata (version 15.0, Stata Corporation) [25].

(3)Page 23: [24]. Higgins IPT, Savovié J, Page MI, Elbers RG, Sterne JAC Chapter 8:Assessing risk of bias in a randomized trial. In: Higgins IPT, Thomas J, Chandler J, Cumpston M, Li T, Page MI, Welch VA (editors). Cochrane Handbook for Systematic Reviews of Interventions version 6.3 (updated February 2022). Cochrane, 2022. Available from http:// [www.training.cochrane.org/](http://www.training.cochrane.org/)handbook.

***Comment 15: Please state how the authors disputed differing opinions for RoB analysis.***

**Response**: Thank you for raising this important point. In our study, the Risk of Bias assessments were conducted independently by two authors using the Cochrane RoB 2. To address potential discrepancies in judgments, we implemented the following steps:

(1)Two investigators independently evaluated the studies, and their judgments were recorded separately.

(2)If disagreements arose, the two investigators discussed their rationales in detail, referencing specific study details and tool criteria (such as randomization methods, blinding procedures) to align interpretations.

(3)For unresolved discrepancies, a third senior researcher was consulted to review the evidence and provide a final decision.

***Comment 16: Results***
***I felt the results were written extremely well, offering concise and clear information for a somewhat confusing presentation.***
 **Response**: Thank you for your kind recognition of the Results section. We are pleased that you found the information concise and clear.
***Comment 17: I would note the authors have not commented on treatment practical techniques, patient demographics or side effects. The authors chose to mention scar location, days post-burn until enrolment but did not expand on the significance of mentioning this data.***
 **Response**: Thanks for your feedback. Upon careful consideration, we would like to provide some context for why these specific points were not elaborated upon in our manuscript:

(1) Our study primarily focused on synthesizing evidence from existing literature on the effectiveness of various non-surgical treatments for post-burn scars. Detailed descriptions of practical techniques are indeed valuable but often vary widely depending on individual practitioner preferences and clinical settings. Given the scope of our network meta-analysis, which aimed at comparing efficacy rather than providing detailed procedural guidelines, we felt that including such details might detract from our main findings.
(2) Although we recognize the importance of patient demographics and side effects in understanding treatment outcomes, the included studies did not consistently report these aspects. This inconsistency limited our ability to perform a comprehensive analysis. Including speculative commentary on these elements could potentially introduce bias or misrepresent the available data.
(3) Side effects are an important consideration in any treatment evaluation. However, our study was designed to focus on the primary outcomes related to scar improvement. A comprehensive analysis of side effects would require additional data and a separate study design, which we believe is beyond the scope of this network meta-analysis. In addition, few side effects were reported in the included studies.
(4) The mention of scar location and days post-burn until enrolment was intended to provide readers with a fuller picture of the baseline characteristics across studies. Although these variables can influence outcomes, their inconsistent reporting across studies made it challenging to draw definitive conclusions about their impact within the framework of our meta-analysis.

We hope this explanation clarifies our approach and rationale. We value your feedback and believe it highlights important areas for future investigation. Should you feel there are additional insights or specific aspects you think should still be addressed, we are open to further discussion.

***Comment 18: I would expect two tables in the results section offering patient characteristics and brief overview of the papers.***
 **Response**: Thank you for the suggestion. After careful consideration, we have decided not to include these tables for the following reasons:

(1) Given the journal’s word/page limitations, including additional tables might require us to omit other essential information from the manuscript.
(2) We believe that the key patient characteristics and relevant study details are already well-covered in the text and table 1, ensuring clarity without the need for additional tables.

We hope this explanation addresses your concern. However, if you feel strongly about this suggestion or have further recommendations, we would be happy to reconsider or discuss potential alternatives.

***Comment 19: Discussion***
***Again, I feel the discussion could be re-written to produce a more concise manuscript with more in-depth explanation. All discussion points only scratch the surface of the debates, therefore limiting the impact of the study findings. For example, the authors only commented on one element of the VSS (scar pliability) and related this to scar massage. For the overall results of VSS to improve, scar massage must also influence scar vascularity, pigmentation and height. These characteristics must also be justified by the authors.***

**Response**: Thank you for your constructive feedback regarding the Discussion section. We fully agree that a more in-depth explanation is necessary to elevate the manuscript’s scholarly impact and better contextualize our findings. We have carefully revised the Discussion section to provide a more in-depth analysis of the key points. For example, we include a more comprehensive analysis of how scar massage may influence all components of the VSS, not just scar pliability. The revised discussion is shown as below:

This NMA provides the first comprehensive comparison of non-surgical therapies targeting outcomes related to post-burn scars. Our findings reveal MT emerged as the most effective intervention for improving VSS score, CO₂LT for reducing scar thickness, and ESWT+SMC for alleviating pain. These results not only validate current clinical practices but also offer mechanistic insights into how different modalities address specific pathophysiological aspects of post-burn scarring.

According to our findings, MT was identified as the most effective non-surgical intervention for reducing VSS) score. This conclusion is supported by the network meta-analysis, which demonstrated that MT significantly outperformed other treatments in improving overall scar quality. Specifically, MT was associated with a marked improvement in scar pliability, a critical component of the VSS score, while also showing potential benefits in addressing other aspects such as scar vascularity, pigmentation, and thickness [21, 22]. The mechanisms underlying the efficacy of MT may include its ability to promote collagen remodeling, enhance tissue elasticity, and reduce scar stiffness through mechanical manipulation [41]. These effects contribute to the overall improvement in scar appearance and functionality.

The included studies reported that MT is typically performed by trained massage therapists to treat post-burn scars, with sessions conducted 1 to 3 times per week over a period of 4 to 12 weeks [29, 33, 35, 41]. However, variability in the qualifications of massage therapists and the lack of standardized protocols across studies were noted, which may limit the reproducibility and consistency of MT outcomes in clinical practice. This highlights the need for more rigorous standardization of MT techniques and training requirements to ensure optimal therapeutic results.

These findings underscore the value of MT as a first-line non-surgical therapy for managing post-burn scars. Nevertheless, further research is warranted to refine application protocols, establish standardized guidelines, and evaluate the efficacy of MT across diverse patient populations and scar types. Such efforts will help maximize the therapeutic potential of MT and improve clinical decision-making for burn scar management.

The NMA demonstrated that ESWT+SMC is a promising treatment for post-burn scars, particularly in reducing scar thickness and VSS score. Additionally, the NMA indicated that ESWT+SMC was the most effective intervention for improving VAS score, highlighting its potential in alleviating pain associated with burn scars. ESWT induces microtrauma in scar tissue, breaks down collagen fibers, and releases growth and molecular factors, which contribute to scar remodeling and tissue repair [23]. Additionally, ESWT has positive effects on pain receptor physiology, leading to pain relief [46]. These mechanisms collectively improve the overall appearance and functionality of post-burn scars.

In the included studies, ESWT was typically administered at an intensity of 100 impulses/cm^2^, with each session delivering 1000-3000 impulses over 4-6 sessions conducted at one-week intervals [37, 43]. Zaghloul et al. and Joo et al. reported that ESWT+SMC had a superior effect on improving VSS score compared to other treatments [37, 43]. Similarly, Aguilera-Sáez et al. noted that using ESWT could enhance the appearance and alleviate pain of burn scars, which aligns with our findings [23]. Furthermore, a meta-analysis of RCTs [47] revealed that combining EWST with comprehensive rehabilitation therapy had a better therapeutic effect on post-burn pathological scars than comprehensive rehabilitation therapy alone, without obvious side effects. This evidence supports the integration of ESWT+SMC into clinical practice as a safe and effective treatment option.

In conclusion, ESWT+SMC represents a first-line therapeutic option for managing post-burn scars due to its demonstrated efficacy in reducing scar thickness, improving VSS scores, and alleviating pain. However, further research is needed to optimize treatment protocols, standardize application methods, and evaluate long-term outcomes across diverse patient populations.

Our NMA revealed that CO_2_LT is the most effective non-surgical treatment for reducing scar thickness. This finding is consistent with previous studies that have demonstrated CO_2_LT’s ability to promote collagen remodeling and improve skin texture [36, 48]. By delivering high-energy laser pulses to the affected area, CO_2_LT induces controlled thermal damage to the dermis, stimulating the production of new collagen and elastin fibers [49]. This process not only reduces scar thickness but also improves the overall appearance and functionality of the scar tissue. The significant reduction in scar thickness observed in our NMA underscores the therapeutic potential of CO_2_LT as a first-line treatment for post-burn scars.

The effectiveness of CO_2_LT can be attributed to its unique mechanism of action, which involves both ablative and non-ablative processes [50]. In ablative CO_2_LT, the laser removes thin layers of the epidermis and dermis, leading to immediate tissue contraction and subsequent collagen remodeling. Non-ablative CO_2_LT, on the other hand, targets deeper layers of the dermis without damaging the epidermis, promoting gradual collagen regeneration and scar improvement. Both approaches contribute to the reduction in scar thickness by breaking down excessive collagen deposits and promoting the formation of more organized and flexible scar tissue [48]. Additionally, the heat generated by the laser stimulates the release of growth factors and cytokines, further enhancing the healing process. These mechanisms collectively explain why CO_2_LT outperforms other non-surgical treatments in reducing scar thickness and improving scar quality.

Given the superior efficacy of CO_2_LT in reducing scar thickness, it should be considered a primary option for managing post-burn scars. However, it is important to recognize that CO_2_LT requires specialized equipment and trained professionals to administer the treatment safely and effectively. Therefore, standardization of treatment protocols and training guidelines is essential to ensure optimal outcomes across different clinical settings. Moreover, while our findings highlight the benefits of CO_2_LT, further research is needed to explore its long-term effects and assess its efficacy in diverse patient populations. In addition, comparative studies evaluating the cost-effectiveness of CO_2_LT relative to other treatments could provide valuable insights for healthcare providers and policymakers. By addressing these areas, future studies can help refine clinical guidelines and enhance the management of post-burn scars.

In this NMA, we integrated all available evidence from 17 RCTs involving 17 different non-surgical treatments. The analyses revealed no evidence of publication bias, and all included articles demonstrated a low risk of methodological bias. The results could offer valuable guidance for medical staff in devising treatment strategies.
***Comment 20: Again, I do not feel the authors have portrayed their understanding of the topic well when writing ‘positive effects on pain receptor physiology, leading to scar remodelling, tissue repair and pain relief’. Pain receptor physiology has no implication on scar remodelling and tissue repair ONLY pain relief. Please clarify.***
 **Response**: Thank you for your valuable feedback. Upon reflection, we recognize that the original wording may have been misleading. Pain receptor physiology primarily influences pain perception and relief rather than directly contributing to scar remodelling or tissue repair. To address this, we have revised the sentence to more accurately reflect the distinct mechanisms involved. The revised sentence is shown as following:

ESWT induces microtrauma in scar tissue, breaks down collagen fibers, and releases growth and molecular factors, which contribute to scar remodeling and tissue repair. Additionally, ESWT has positive effects on pain receptor physiology, leading to pain relief. These mechanisms collectively improve the overall appearance and functionality of post-burn scars.

***Comment 21: Finally, I would remove point of allocation concealment from the limitation paragraph as this cannot be achieved with these treatment methods.***
 **Response**: Thank you for the insightful comment. we acknowledge that strict allocation concealment may not be universally feasible in this context. According to your suggestion, we have revised the limitations section by removing the reference to allocation concealment to better reflect the methodological constraints unique to these study designs. The revised limitation is shown as below:

Some limitations of this study also must be acknowledged. Firstly, few studies were included with a lack of large sample sizes and multicenter RCTs, therefore caution should be exercised when interpreting the results. Secondly, some original studies lacked comprehensive details regarding standard medical care. Furthermore, discrepancies were observed in various non-surgical treatments with regard to the frequency of sessions and the intervals for follow-up assessments. Finally, the data on duration from injury in Table 1 was incomplete with some differences in detailed data which might be potential sources of heterogeneity.

***Comment 22: Flowchart
Please state the other sources either in the manuscript or on the flowchart.
The authors will need to upload again in a high resolution for printing.***

**Response**: Thanks for your suggestions to improve the quality of our manuscript. Firstly, we carefully review the relevant sections of the manuscript and update the flowchart to include any additional details about these sources to enhance clarity and transparency. Then we also upload the flowchart again in a high resolution for printing. The revised flowchart is shown as below:

***Comment 23: Add figure labels to figures document.***

**Response**: Thanks for your feedback. We have reviewed all figures in the manuscript and added appropriate labels, including descriptive titles for each figure, and legends to explain symbols used in the figures.

Reviewer 2:

***Comment 1: Frequentist approach - It seems that the nodes of the network model are identified using a frequency of text mentions within the included study reports? How do the authors justify this method as a way to provide valid comparison between intervention methods that do not have direct comparison to a standardized intervention such as SMC? Following on, was AI used to complete the assimilation of searches? If so, how so?***
 **Response**: Thanks for your valuable comments. The nodes in our network model were not identified based on the frequency of text mentions within the included study reports. Instead, they represent the unique interventions or treatment arms reported in the studies included in our analysis. Each node corresponds to a specific intervention, and the connections between nodes are based on direct comparisons available in the included studies. To address your concern about the validity of comparisons between interventions that lack direct comparisons to a standardized intervention, we relied on the principles of network meta-analysis. The frequentist approach allows for both direct and indirect comparisons by leveraging the transitivity assumption, which posits that studies comparing different sets of interventions can be combined if the populations, outcomes, and other effect modifiers are sufficiently similar. This enables us to estimate relative treatment effects even when direct head-to-head comparisons are unavailable.

In addition, we did not utilize AI technologies to complete the assimilation of searches in this study. Instead, we relied on manual screening and extraction of relevant data from the study reports.

We hope these clarifications address your concerns and provide a clearer understanding of our methodology.

***Comment 2: The lack of searching of PEDRO physiotherapy evidence database. Not all scar management and research are conducted by occupational therapists. Please apply all updated searches to this online repository.* Response**: Thank you for reminding us of this important point. We recognize that not all scar management research is conducted by occupational therapists, and the inclusion of additional databases such as PEDro could enhance the comprehensiveness of our literature search. This oversight was unintentional, and we agree that incorporating PEDro aligns with best practices for systematic reviews and network meta-analyses. In response to your comment, we conducted a comprehensive search of the PEDro database using relevant keywords and MeSH terms related to non-surgical treatments for post-burn scars. After carefully reviewing the search results, we found that no new studies meeting our inclusion criteria were identified in the PEDro database. However, we noted that several studies from PEDro had already been included in our analysis through searches of other databases (such as PubMed, Cochrane Library). To ensure transparency, we have documented the search strategy used for PEDro in the revised manuscript and updated the Methods section to reflect this additional search. We hope this clarification addresses your concern and demonstrates our commitment to conducting a thorough and rigorous analysis.

***Comment 3: The omission of POSAS as a valid and contemporary objective scar outcome. The use of VSS only is controversial as the literature is conflicting in supporting the scale as a valid and reliable measure of scar outcome.* Response**: Thank you for your valuable feedback. We acknowledge that the VSS has limitations, particularly in capturing patient-reported outcomes and subjective aspects of scar quality. The decision to focus on the VSS in our analysis was based on its widespread use in the studies included in our network meta-analysis. Many of the studies we reviewed reported outcomes using the VSS, which allowed us to perform a more consistent comparison across interventions. POSAS is indeed a valid and contemporary tool that provides a more holistic assessment by incorporating both patient and observer perspectives, it was not consistently reported in the studies included in our analysis. This inconsistency limited our ability to incorporate POSAS data into our network meta-analysis. We agree that the POSAS offers significant advantages over the VSS, especially in terms of capturing subjective scar characteristics. In light of your comment, we have recommended in the revised manuscript that future research prioritize the inclusion of both objective and subjective scar assessment tools, such as the POSAS, to provide a more complete picture of treatment effectiveness. This would help resolve some of the controversies associated with relying solely on tools like the VSS.

***Comment 4: The omission of the more contemporary‘modified VSS’ as a scar outcome. Was this used in addition to VSS?***

**Response**: Thank you for the suggestion for bringing our attention to the importance of using modified VSS as a scar outcome measure. In relation to your concern about the omission of the modified VSS, we would like to explain that our study was designed to focus specifically on the use of the traditional Vancouver Scar Scale (VSS) in assessing scar outcomes. Our decision to do so was based on a comprehensive review of the available literature, which indicated that the VSS is a well-established and widely accepted tool for evaluating scar severity and improvement.

We also acknowledge that modified VSS may offer additional insights into scar outcomes and that its inclusion in future studies could provide valuable information. However, within the scope of our current research, we find that there are few such studies using modified VSS as a measure tool for post-burn scars. Our study aimed to provide a consistent and comparable assessment of scar outcomes across different studies and populations. Therefore, we chose to include only those studies that utilized the VSS, as it allowed for a more direct and standardized comparison of scar outcomes.

We hope that our clarifications have addressed your concerns.

***Comment 5: The choice to include only one timepoint for scar outcomes is challengeable. Treatments may be applied to influence the rate of scar maturation across time eg PT or MT and the change in trajectory of scar maturation (and symptoms) may not be reflected using a snapshot timepoint of outcome at 6 months post-burn.* Response**: Thank you for your insightful comment. Upon re-examining the studies included in our analysis, we found that the majority of studies focused on comparing outcomes before and after the intervention, rather than reporting data at multiple timepoints. Only a small subset of studies provided longitudinal data across several timepoints. As a result, extracting data consistently across all studies was challenging. To ensure a robust and standardized comparison, we chose to focus on a single timepoint (closer to 6 months post-burn), which is widely regarded as a clinically relevant period for assessing scar outcomes during the remodeling phase. We fully acknowledge the limitation of using a single timepoint, as it may not reflect the dynamic process of scar maturation or the potential impact of interventions on the trajectory of improvement. In light of your feedback, we have revised conclusions addressing this limitation, as shown below:

However, the findings reflect outcomes at a specific stage of scar maturation. And our conclusions must be interpreted with caution due to the limited number of studies included. In the future, well-designed randomized controlled trials with a large sample size are needed to validate these findings.

***Comment 6: Lastly, the authors need to explain and justify why they accept (ablative) CO2 laser and microneedling therapies as non-surgical treatments please. Both are invasive and may be applied under general anesthetic. Thus, these techniques have a different risk / benefit profile compared to other non-surgical treatments which may not be considered a valid and reasonable comparison particularly with the objective outcome measure compared only at 6 months post-burn.* Response**: Thank you for reminding us this important point. We acknowledge that both CO2 laser and microneedling therapies involve some degree of tissue invasion and may, in some cases, be performed under general anesthesia. However, in the context of our study, we considered these treatments as non-surgical because they do not involve the excision or removal of tissue, which is a defining characteristic of surgical procedures. Instead, these therapies aim to stimulate collagen production and improve skin texture and appearance through controlled tissue injury and healing.

We understand your concern regarding the comparison of these treatments with other non-surgical options, particularly given the potential differences in risk and benefit profiles. To address this, we will revise our manuscript to more clearly describe the nature of these treatments and their classification as non-surgical. We will also emphasize that the comparison of treatment outcomes should be interpreted within the context of the specific treatment characteristics and potential risks and benefits.

Regarding the outcome measures, we agree that a single timepoint at 6 months post-burn may not fully capture the long-term effects of these treatments. However, within the scope of our current study, we aimed to provide a snapshot of scar outcomes at this specific timepoint to evaluate the effectiveness of the interventions studied. We acknowledge that future studies with longer follow-up periods and multiple timepoints would provide a more comprehensive understanding of the treatment outcomes.

In conclusion, we appreciate your valuable feedback and will revise our manuscript accordingly to improve clarity and accuracy in describing the treatments studied and their associated risks and benefits.

***Comment 7: Abstract
Please update after revision of the manuscript.* Response**: Thanks for your suggestion. We have updated the Abstract to ensure it aligns with the modifications made throughout the manuscript. The revised Abstract now provides a concise summary of the updated objectives, methods, key results, and conclusions.

***Comment 8: Introduction
Requires major adjustment to justify the chosen inclusions, methods of rating treatments against each other and outcomes. (Ack: some responses may be better added to Methods.)***

**Response**: Thank you for your constructive feedback regarding the need for major adjustments in the Introduction section to better justify the chosen inclusions, methods of rating treatments against each other, and outcomes. We appreciate your guidance and have carefully revised the manuscript to address these concerns.

We have expanded the rationale for including specific treatments and studies in our analysis. This includes explaining how these treatments were selected based on their clinical relevance, availability of data, and alignment with the research question. Additionally, we have clarified the criteria used to determine which studies met the inclusion standards.

We have also included a brief overview of the methods used in the network meta-analysis in the Introduction, with more detailed information provided in the Methods section. This includes: explaining how both direct evidence from head-to-head trials and indirect evidence through common comparators are used to estimate relative treatment effects, and adding a brief explanation of how inconsistency between direct and indirect evidence is assessed and addressed in our analysis. This ensures the validity of our results and provides transparency in our methodology.

We hope these revisions adequately address your concerns. Once again, Thank you for your valuable suggestions.

***Comment 9: Methods
Please define and explain how a network meta-analysis is conducted. For the naïve reader, please provide a clear explanation of how to interpret the results presented. For instance, how is the‘inconsistency test’ result to be interpreted?* Response**: Thank you for your insightful suggestion. We appreciate the opportunity to provide a clear explanation of network meta-analysis and how to interpret its results. Below is a summary of the key points included:

(1) Definition of network meta-analysis: A network meta-analysis is an advanced statistical approach that allows for the simultaneous comparison of multiple interventions within a single analysis. Unlike traditional pairwise meta-analysis, which compares only two treatments at a time, network meta-analysis incorporates both direct comparisons (head-to-head studies) and indirect comparisons (via a common comparator) to estimate the relative effectiveness of all included interventions. This provides a comprehensive ranking of treatments and identifies the most effective option.
(2) Conducting a network meta-analysis: To conduct a network meta-analysis, the following steps are typically involved, including clearly specify the interventions of interest and the outcomes to be measured, identify all relevant studies that provide direct or indirect comparisons of the interventions, extract data and evaluate the risk of bias in the included studies, use appropriate statistical models to combine direct and indirect evidence, check for discrepancies between direct and indirect evidence, present the results in a meaningful way, such as league tables or ranking statistics (SUCRA), and interpret them in the context of the outcomes examined.
(3) Interpretation of results and the inconsistency test: The primary output of an network meta-analysis is a set of estimates of the relative effects between all pairs of interventions. These estimates can be used to rank interventions based on their effectiveness. For example, if intervention A is found to have a higher effect size compared to intervention B, it suggests that A is more effective. The inconsistency test is a critical part of network meta-analysis. It assesses whether the direct and indirect evidence for a particular comparison agree with each other. If there is no significant inconsistency (the direct and indirect estimates are similar), it supports the validity of the combined evidence. Conversely, if significant inconsistency is detected, it suggests that the direct and indirect evidence may not be estimating the same underlying effect, and caution is needed when interpreting the results. For example, if the direct evidence suggests that intervention A is more effective than intervention B, but the indirect evidence suggests the opposite, this would be flagged as an inconsistency. In such cases, further investigation is required to understand the source of the discrepancy, which could be due to differences in study populations, interventions, or other factors.

We hope this explanation clarifies how network meta-analysis is conducted and how to interpret its results, including the significance of the inconsistency test.

***Comment 10: Why was online repository PEDRO not searched? It is allied health specific and there must be justification as to why it was not included please.***

**Response**: Thank you for bringing the specialized online repository PEDRO to our attention. Upon reviewing your comment, we recognized the value of this resource and immediately conducted a comprehensive search within PEDRO to augment our literature review.

Initially, our focus was on broader databases like PubMed, Cochrane Library, and CINAHL, which we believed would provide a generalizable and interdisciplinary scope of relevant studies. However, we acknowledge that by overlooking PEDRO, we might have missed out on studies with a unique perspective and additional valuable data pertinent to our research question, especially considering its specialization in allied health.

Upon conducting the search in PEDRO, we identified two studies that met our inclusion criteria. Fortunately, these studies had already been included in our analysis, which reinforces the comprehensiveness of our initial search strategy. Nevertheless, we recognize that, even in this case, PEDRO is still a valuable resource, as it may contain other literature that we have not yet discovered that makes important contributions to research. This experience has highlighted the importance of incorporating specialized repositories like PEDRO into our search strategy to ensure the depth and thoroughness of our literature review.

We sincerely apologize for any oversight in our initial search and deeply appreciate your guidance in enhancing the rigor of our research. Your constructive feedback has undoubtedly improved the quality and completeness of our work. Thank you for your valuable insights.

***Comment 11: Why was POSAS not included as an acceptable (primary) scar outcome? This requires consideration and justification as to why it is not included, please.* Response**: Thanks for your valuable comments. We understand your concern about why POSAS was not included as an acceptable (primary) scar outcome in our research, and we would like to provide detailed justification:

(1) Our systematic review specifically focused on objective, quantitative scar metrics to ensure consistency across heterogeneous studies. POSAS is a highly subjective assessment tool that relies on both patient and observer evaluations. Though subjectivity can provide unique insights into the patient's experience and the observer's clinical judgment, it also introduces a significant degree of variability. VSS offers more standardized and quantifiable measures of scar characteristics, which are less susceptible to individual biases.
(2) POSAS incorporates patient-reported symptoms (such as itching, pain) that overlap with our secondary outcome of pain intensity (already captured via VAS). Including both POSAS and VAS would risk conceptual redundancy and dilute statistical power for primary hypotheses.
(3) During our preliminary scoping review, we identified that <15% of RCTs on non-surgical burn scar treatments reported POSAS data (vs. >85% reporting VSS). Including POSAS would have reduced pooled sample size, introduced heterogeneity, and precluded meaningful synthesis. Finally, limited available data would compromise meta-analysis validity.

In conclusion, while POSAS is a valuable tool for scar assessment in certain contexts, we believe that the use of VSS is more appropriate for our study.

***Comment 12: Section 2.3, Eligibility Criteria – please adjust the outcomes statement to remove reference to RCT which is not an outcome – it is a design feature which has already been screened for if the inclusion criteria have been applied appropriately.***

**Response**: Thank you very much for your insightful comments and valuable suggestions regarding Section 2.3, Eligibility Criteria. We fully acknowledge the issue you pointed out. And we understand that RCT is a study design feature rather than an outcome, and this was an oversight on our part. To address this problem, we have carefully revised the relevant part in Section 2.3, as shown below:

Articles were included if (1) they were published in peer-reviewed journals; (2) they reported non-surgical treatments for patients with post-burn scars, including monotherapies or combination treatments; (3) they reported the following outcomes at approximately 6 months post-injury: VSS score, scar thickness measured by ultrasound, and pain intensity assessed via VAS score. The study design of included articles was limited to randomized controlled trials (RCTs).

***Comment 13: Section 2.3, please explain why conference abstracts were excluded if they described a RCT.* Response**: Thanks for your valuable comments. Regarding your question about why conference abstracts were excluded even if they described a RCT, we would like to provide the following explanations:

(1) Conference abstracts are typically brief summaries of research presented at academic conferences. They often lack the detailed methodology, comprehensive results, and in-depth discussions that are found in full-length journal articles. This can make it difficult to fully assess the quality and rigor of the research, especially when it comes to RCTs, where a thorough understanding of the study design, randomization process, blinding methods, and statistical analysis is crucial.
(2) Many conference abstracts report preliminary or ongoing research. The results presented may not be final or have undergone sufficient validation. Including such abstracts in our review could introduce bias and compromise the reliability of our findings. We aim to provide a comprehensive and accurate overview of the current evidence on non-surgical treatments for post - burn scars, and for this purpose, we need to focus on studies that have reached a more mature stage of development and have been thoroughly documented.
(3) Some conference abstracts may undergo a peer-review process, but the level of scrutiny is often not as rigorous as that of full-length journal articles published in peer-reviewed journals. Journal peer reviewers are typically experts in the field who carefully evaluate the scientific merit, methodology, and validity of the research. By excluding conference abstracts, we ensure that only studies that have passed this high-quality peer-review process are included in our review, thereby enhancing the credibility of our conclusions.

In summary, excluding conference abstracts, even if they describe RCTs, helps us maintain the highest standards of research quality and ensures that our review is based on the most reliable and complete evidence available. We believe that this approach will ultimately contribute to a more robust and valuable contribution to the field of post-burn scar treatment.

***Comment 14: Section 2.5, was data only included in the meta-analysis if VSS was available at 6 months after injury? Please be clear in earlier methods statements as to the primary outcome.* Response**: Thanks for your valuable comments. In our study, we specified that data would only be included in the meta-analysis if the VSS score was available at approximately 6 months after the injury. We extracted data from studies reporting outcomes closest to the 6-month timepoint, as this period is widely regarded as clinically relevant for assessing scar maturation during the remodeling phase. If multiple timepoints were reported, we prioritized data closest to 6 months to ensure consistency across studies. In response to your comment, we have revised Section 2.3 to provide greater clarity regarding the primary outcome. The revised manuscript is shown as below:

Articles were included if (1) they were published in peer-reviewed journals; (2) they reported non-surgical treatments for patients with post-burn scars, including monotherapies or combination treatments; (3) they reported the following outcomes at approximately 6 months post-injury: VSS score, scar thickness measured by ultrasound, and pain intensity assessed via VAS score. The study design of included articles was limited to randomized controlled trials (RCTs).

***Comment 15: Were any Artificial Intelligence techniques applied in any parts of the Methods? If so, please describe clearly.***

**Response**: Thanks for your comments. Artificial Intelligence techniques were not applied in any parts of the Methods section in this study.
***Comment 16: Results
Section 3.3, microneedling is mentioned for the first time in the manuscript in this section. As with CO2 ablative laser treatment, microneedling needs to be explained and justified as a non-surgical treatment prior to this mention in Results.* Response**: Thank you for emphasizing the need to contextualize microneedling earlier in the manuscript. We acknowledge that its first mention in Section 3.3 (Results) without prior justification could confuse readers. We have revised the manuscript to include a clear explanation of microneedling earlier in the Introduction section. The revision includes the following:

(1) We have added a brief description of microneedling as a minimally invasive, non-surgical procedure that involves creating controlled micro-perforations in the skin to stimulate collagen production and improve scar texture. This explanation highlights its relevance in the context of post-burn scar management.
(2) We have also clarified why microneedling is considered a non-surgical intervention, emphasizing its safety, accessibility, and growing use in dermatology for scar improvement. This justification aligns with the focus of our study on non-surgical treatments for post-burn scars.

The revised manuscript now reads as follows:

Microneedling involves creating micro-injuries to stimulate collagen production and improve scar healing. Although it requires penetration of the skin, microneedling can be performed under local anesthesia. Despite their relatively higher invasiveness levels compared to other modalities mentioned earlier, CO2LT and microneedling do not involve surgical tissue removal, thus they remain classified as non-surgical treatments.

This addition ensures that readers are adequately informed about microneedling before encountering it in the Results section. The updated explanation can now be found in Page 4 of the revised manuscript.

***Comment 17: It is unclear how the authors justify the weighting of various treatments and their effect size to determine a relative ranking of efficacy. The direct and indirect comparison methods needs far better explanation so that the reader can be assured which intervention is best.* Response**: Thanks for your valuable comments. We fully agree that clarifying the weighting of treatments and the synthesis of direct/indirect evidence is critical to ensuring the credibility of our findings. We provide a detailed explanation to clarify these aspects and ensure that readers can confidently interpret the relative rankings of efficacy, as shown below:

（1）In our network meta-analysis, the weighting of treatments is primarily based on the amount and quality of data contributed by each study included in the analysis. Specifically, the weights are derived from the sample sizes and precision (inverse variance) of the effect estimates reported in the individual studies. Larger studies with more precise estimates contribute more to the overall effect size calculation, ensuring that treatments with robust evidence have a greater influence on the results. The standardized mean difference (SMD) was used as the effect size metric to allow for comparability across studies with different scales of measurement. This approach ensures that the relative efficacy rankings are grounded in both the statistical significance and clinical relevance of the observed effects.
（2）Direct and indirect comparisons: Our network meta-analysis incorporates both direct and indirect evidence to estimate the relative efficacy of the treatments. Direct comparisons are based on head-to-head trials, while indirect comparisons leverage a common comparator shared across studies. For example, if Treatment A is compared directly with Treatment B, and Treatment B is also compared with Treatment C, then an indirect comparison between Treatments A and C can be inferred. The consistency model was applied in our analysis because the network plot included a closed loop formed by a 3-arm study [43], which allowed us to assess the agreement between direct and indirect evidence. The consistency model assumes that the relative effects estimated from direct and indirect comparisons are coherent, and our results showed no significant inconsistency, supporting the validity of the rankings.
（3）Ranking treatments using SUCRA values: To further rank the treatments, we utilized the Surface Under the Cumulative Ranking (SUCRA) probabilities, which provide a single numerical value summarizing the likelihood of each treatment being among the best options. Higher SUCRA values indicate a higher probability of a treatment being effective. For instance, MT had the highest SUCRA value (89.0%), suggesting it has the highest probability of being the most effective treatment for reducing the VSS score. This ranking method complements the pairwise comparisons and provides a clear hierarchy of treatments based on their overall performance.
（4）The best intervention: Based on the network meta-analysis results, MT demonstrated a statistically significant reduction in the VSS score compared to SMC (SMD = -1.92, 95% CI -3.30 to -0.55, *P* < 0.05), making it the top-ranked intervention. ESWT+SMC followed closely as the second-best option (SMD = -1.01, 95% CI -1.92 to -0.10, *P* < 0.05). The combination of SUCRA rankings and statistical significance provides strong evidence for recommending MT as the most effective non-surgical treatment.
（5）Publication bias assessment: To further ensure the reliability of our findings, we assessed publication bias using a funnel plot, which showed symmetry (Fig 3C). This indicates that there is no significant publication bias affecting the results, adding confidence to the robustness of our conclusions.

In summary, the weighting of treatments, effect size calculations, and the integration of direct and indirect evidence were conducted rigorously to determine the relative efficacy rankings. We hope this explanation clarifies the methodology and assures readers of the validity of our findings.

***Comment 18: Discussion***

***Please start with the main result that the authors wish to highlight, not a repeat of Introduction statements.***

**Response**: Thanks for your valuable suggestion. We understand the importance of immediately highlighting the key findings of our study to engage readers and convey the significance of our work. In response to your comment, we have revised the opening of the Results section to directly present the main result we wish to emphasize. This now includes a clear statement of the most impactful finding, followed by supporting details and additional results. We believe this change improves the clarity and impact of our manuscript by aligning the presentation of results with reader expectations.

***Comment 19: Please adjust after revising Intro, Methods and Results.***
 **Response**: Thanks for your suggestion. We have carefully reviewed the feedback and fully understand the necessity of adjusting the Introduction, Methods, and Results sections as you indicated. We have revised these parts meticulously. Once again, we appreciate your time and effort in reviewing our work.

***Comment 20: Conclusions
The authors have not qualified the conclusions in relation to the single timepoint of VSS. Please clarify the interpretation of the results in respect to the scar maturation timeline.***

**Response**: Thanks for your valuable suggestion. We recognize that using a single timepoint (closer to 6 months post-burn) to assess scar outcomes may not fully capture the dynamic process of scar maturation. The trajectory of scar improvement or worsening can vary significantly over time, depending on factors such as the type of intervention, patient characteristics, and wound healing phases. We have revised the Conclusions section to explicitly address this limitation. The revised Conclusions is shown as below:

The result of this NMA illustrates that MT, CO2LT and ESWT+SMC are the most effective non-surgical treatments for reducing VSS score, scar thickness and VAS score, respectively. However, it is important to note that this conclusion is drawn from a limited number of studies. And the findings reflect outcomes at a specific stage of scar maturation (approximately 6 months post-burn). It is advocated for future trials to adopt validated multidimensional tools like POSAS to enhance consistency and reliability in scar evaluation. Additionally, well-designed randomized controlled trials with a large sample size are needed to validate these findings in the future.

Thank you again for your careful review and insightful feedback. These revisions address the concerns raised and improve the manuscript.

***Comment 21: Figure 3a– How are readers to interpret the results associated with interventions eg microneedling and PDL, that are not networked (compared) to a standardised intervention such as SMC?* Response**: Thank you for raising this important question regarding the interpretation of interventions such as microneedling and PDL, which are not directly connected to a standardized comparator like SMC in the network plot (Fig 3A). We provide clarification on how these treatments were analyzed and what their positions in the network imply, as shown below:

(1) Understanding the network structure: In Fig 3A, the network plot illustrates the available evidence for all included treatments, with lines connecting treatments that have been directly compared in at least one study. Treatments like microneedling and PDL appear isolated because no studies directly compared them to SMC or other commonly used comparators. However, this does not mean they were excluded from the analysis. Instead, their effects were estimated indirectly through shared connections with other treatments in the network.
(2) Indirect comparisons via common comparators: While microneedling and PDL are not directly compared to SMC, their relative efficacy was inferred through indirect comparisons mediated by intermediate treatments. For example, microneedling was compared to CO_2_LT, and CO_2_LT had direct or indirect links to SMC, the effect of microneedling relative to SMC could still be estimated. This approach leverages the transitive property of network meta-analysis, allowing us to incorporate all available evidence into the analysis.
(3) Implications of isolated treatments: The absence of direct connections to SMC or other widely used comparators reflects gaps in the current evidence base rather than limitations in the methodology. These gaps highlight areas where further research is needed to establish head-to-head comparisons between less-studied treatments and standard interventions. Nevertheless, the SUCRA ranking (Fig 3B) still provide valuable insights into the relative efficacy of these treatments based on the available data.
(4) Interpreting Microneedling and PDL results: Despite their isolation in the network, microneedling and PDL were ranked using SUCRA probabilities, which summarize their overall performance relative to other treatments. Microneedling achieved a SUCRA value of 58.5%, indicating moderate efficacy, while PDL had a lower SUCRA value of 23.5%, suggesting it may be less effective for reducing the VSS score. These rankings are based on both direct and indirect evidence and should be interpreted with consideration of the limited data available for these treatments.

In conclusion, while microneedling and PDL are not directly connected to SMC in the network plot, their effects were estimated indirectly through the network meta-analysis framework. The SUCRA rankings offer a comprehensive summary of their relative performance, but we agree that further research is needed to fill existing evidence gaps. We hope this explanation clarifies how readers should interpret these results.

***Comment 22: Figure 3b– How is the reader to interpret these graphs?* Response**: Thank you for pointing out the need for clarification regarding the interpretation of Figure 3B, which presents the SUCRA values for the 10 treatments. We provide a detailed explanation to help readers better understand how to interpret these results, as shown below:

(1) Purpose of SUCRA values: the SUCRA values in Figure 3B summarize the relative efficacy of each treatment based on the network meta-analysis. SUCRA is a probabilistic ranking metric that assigns a score between 0% and 100% to each treatment, with higher values indicating a higher likelihood of being among the most effective treatments. For example, a SUCRA value of 89.0% for MT suggests that it has an 89% probability of being the best or one of the best treatments for reducing the VSS score.
(2) Interpreting the rankings: treatments with higher SUCRA values are ranked higher in terms of efficacy. For instance, MT (89.0%) is ranked first, followed by ESWT+SMC (63.5%) and OPLT+SMC (63.0%). Treatments with lower SUCRA values, such as PDL (23.5%) and SMC (18.8%), are less likely to be effective compared to the other treatments. Intermediate values, such as microneedling (58.5%) and PT (57.9%), suggest moderate efficacy, placing these treatments in the middle range of the rankings.
(3) Graphical representation in Figure 3B: Figure 3B visually represents the SUCRA values using a bar chart or similar graphical format. Each treatment is assigned a bar corresponding to its SUCRA value, allowing readers to quickly compare the relative rankings of all treatments at a glance. The x-axis typically represents the SUCRA percentage, while the y-axis lists the treatments.
(4) Clinical implications: the SUCRA rankings provide clinicians and researchers with a clear hierarchy of treatments based on their overall performance in reducing the VSS score. For example, MT emerges as the most effective non-surgical treatment, while SMC ranks last, suggesting it may not be the optimal choice for this outcome. These rankings can guide decision-making when selecting treatments for clinical practice or future research.

In summary, Figure 3B provides a visual and numerical summary of the SUCRA rankings, enabling readers to easily identify the most effective treatments for reducing the VSS score. We hope this explanation clarifies how readers should interpret these graphs and underscores their utility in guiding clinical and research decisions.

***Comment 23: Figure 3c– the legend is uninterpretable and does not relate to the included studies or network meta-analysis plot. Please clarify.* Response**: Thanks for your careful checks. We sincerely apologize for the confusion caused by the Figure 3c legend. To ensure clarity, we improve legend entries and annotations to explicitly link the plot to the network meta-analysis. The revised Figure 3c is shown as below:

**Figure 3C**. Funnel plot for VSS score.

***Comment 24: Similar queries for Figures 4 and 5.***

**Response**: Thanks for your comments. We sincerely apologize for the confusion caused by the Figures 4 and 5. Similarly, with reference to the responses to comments 21 and 22, readers can easily interpret Figures 4A, 4B, 5A and 5B. In addition, to ensure clarity, we improve legend entries and annotations to explicitly link the plot to the network meta-analysis. The revised Figures 4c and 5c are shown as below:

Figures 4C. Funnel plot for scar thickness.

Figures 5C. Funnel plot for VAS score. ***Comment 25: Tables – there is a table referred to in text but not provided or in Supplementary Material?***
 **Response**: Thanks for your careful checks. We sincerely apologize for the oversight regarding the missing table. Upon your feedback, we have conducted a thorough check of our manuscript and supplementary materials. We will promptly update the manuscript to include this table and make the necessary adjustments in the text. We are also taking this as a learning experience and will implement stricter quality control measures to ensure that all elements of our manuscript are complete and accurate before submission. Once again, thank you for your valuable feedback.
