# Supplementary material for: Non-surgical treatments for post-burn scars: A network meta-analysis
Source: PLoS One. 2025 Aug 21;20(8):e0330428. doi: 10.1371/journal.pone.0330428 (PMC12370048; doi:10.1371/journal.pone.0330428)
Supplement: Attachment — Submitted filename: Response_to_Reviewers_auresp_2.docx [file pone.0330428.s006.docx]

Dear Editors and Reviewers,

Thank you very much for giving us opportunities to revise our manuscript, and we appreciate the reviewer a lot for his positive and constructive comments and suggestions. We have studied reviewer’s comments carefully and have made revisions. We hope the corrections will meet with your approval.

Reviewer 1:

***Comment 1: POSAS – the authors’ arguments about POSAS exclusion are weak. They demonstrate that the authors do not appreciate the subjectivity and ambiguity of the scoring of the VSS. The limitations of the VSS were the very premise of and impetus to develop the POSAS as an alternative scar outcome measure. To further exclude POSAS on the basis of redundancy with VAS, also suggests a lack of understanding of the foibles of groupwise ‘quantitative’ pain measures. That said, if the authors are unable to extend the scope of this study (and NMA) then please add text to affirm the direction that future reviews of this type and, or studies should be going.***

**Response**: Thank you for your insightful comments and suggestions. We fully acknowledge that VSS has limitations due to its subjective nature and potential ambiguity in scoring. Indeed, these limitations were among the key motivations for the development of more comprehensive and patient-centered outcome measures such as POSAS. We appreciate your emphasis on this point and agree that tools like POSAS provide a more holistic evaluation of scar characteristics by incorporating both observer and patient-reported outcomes.

In our review, we excluded POSAS not because of assumed redundancy with other scales, but due to insufficient data availability across the included studies. Many trials did not report outcomes using POSAS, which limited its feasibility as an outcome measure for our NMA. We strongly agree with your suggestion that future reviews and clinical studies should aim to incorporate more patient-centered and multidimensional tools like POSAS to better reflect the complexity of post-burn scar assessment.

We also appreciate your clarification regarding the limitations of pain measures such as VAS, and your concern about potentially conflating them with broader outcome scales like POSAS. While VAS was selected for this analysis due to its widespread use and availability across studies, we recognize that it captures only one dimension of the patient experience and cannot substitute for more comprehensive assessments.

To address your concerns, we have expanded the Discussion section to explicitly acknowledge the limitations of relying primarily on VSS and VAS, and to advocate for the inclusion of validated, multidimensional tools such as POSAS in future research. The expanded text is shown as below:

In this NMA, though Our analysis focused on commonly reported quantitative outcomes such as VSS and VAS scores, we acknowledge the limitations of these scales in capturing the full spectrum of scar quality and patient experience. The POSAS, which integrates both observer and patient perspectives on multiple scar attributes, offers a more comprehensive alternative. Future studies and systematic reviews should prioritize the use of standardized, multidimensional tools such as POSAS to enhance the validity and clinical relevance of outcome reporting in post-burn scar management.

***Comment 2: How do the authors reconcile that by excluding the studies using the more contemporary POSAS outcome measure also more than likely excludes the more contemporary scar treatments? The authors confirm that <15% of potentially eligible studies reported POSAS results which is an indication of the point made above, and there is a likely bias introduced to the NMA by excluding more recent studies as per the review methodology.***

**Response**: Thank you for raising this important point. We fully agree that excluding studies reporting outcomes using POSAS may also result in the omission of more recent trials employing newer or more advanced scar treatments.

Although We recognize the value of POSAS as a comprehensive and patient-centered outcome measure, this NMA was constrained by the availability and consistency of reported data across studies. The majority of published trials still rely on VSS and VAS due to their widespread use and ease of interpretation in clinical settings. Given the limited number of studies reporting POSAS outcomes, including it as an outcome measure would have significantly reduced the pool of usable data and compromised the robustness and generalizability of our analysis.

We also acknowledge that this decision introduces a potential selection bias and limits our ability to capture findings from more contemporary studies. Therefore, we have added a statement in the Limitations section of our manuscript to explicitly address this issue:

In addition, one limitation of this NMA is the exclusion of studies reporting only POSAS outcomes, which may have led to the omission of more recent interventions that adopt this more comprehensive and patient-centered assessment tool. This highlights the need for future studies to harmonize outcome reporting and adopt standardized, multidimensional scales such as POSAS to enhance the comparability and relevance of evidence in post-burn scar management.

***Comment 3: Lastly, after the comments made by multiple reviewers, why were sensitivity analyses not conducted in this revision? In particular, why were studies with possible or confirmed application of (ablative) CO2 laser and microneedling therapies under general anaesthetic, not removed from the NMA corpus to confirm the effect size of these techniques in a way that is much more comparable to other non-surgical techniques with a far lower risk and complication profile? The invasive techniques and general anaesthetic have much more pronounced risk compared to other non-surgical treatments and this issue of relative benefit must be explored so that readers can make an informed decision as to the interpretation of results for their practice.* Response**: Thank you for your insightful comments and for highlighting the importance of addressing potential biases in our NMA of non-surgical treatments for post-burn scars. We understand the importance of conducting sensitivity analyses to assess the robustness of our findings. However, we did not perform additional sensitivity analyses in this revision for the following reasons:

(1) data availability and study characteristics: the number of studies included in our NMA was limited, and removing studies with possible or confirmed application of ablative CO_2_ laser and microneedling therapies under general anaesthetic would have significantly reduced the sample size. This could have compromised the statistical power and reliability of the results.
(2) clinical heterogeneity: though ablative CO_2_ laser and microneedling therapies under general anaesthetic are indeed more invasive and carry higher risks compared to other non-surgical treatments, they also represent a distinct subset of interventions within the broader category of non-surgical treatments for post-burn scars. Excluding these studies might have introduced bias by omitting important clinical scenarios.

In this NMA, we conducted a series of consistency checks to assess the agreement between direct, indirect, and network estimates. As shown in the tables, the P-values for all comparisons were greater than 0.05, indicating no statistically significant differences between direct and indirect estimates. This suggests good consistency within our NMA, which supports the reliability of our findings.

**Table 1. The result of nodesplit in VSS.**

| side | Direct | |  | Indirect | |  | Difference | | |  | tau |
| --- | --- | --- | --- | --- | --- | --- | --- | --- | --- | --- | --- |
|  | coef. | Std.Err |  | coef. | Std.Err |  | coef. | Std.Err | *P* >∣Z∣ |  |  |
| CO_2_LT VS CO_2_LT+PDL | 0.000014 | 0.7001207 |  | -1.459051 | 1.842309 |  | 1.459065 | 1.964455 | 0.458 |  | 0.6305058 |
| CO_2_LT VS Microneedling | _ | _ |  | _ | _ |  | _ | _ | _ |  | _ |
| CO_2_LT VS PDL | -0.0281351 | 0.7111481 |  | 1.431045 | 1.829667 |  | -1.459198 | 1.9645 | 0.458 |  | 0.6305058 |
| CO_2_LT VS RT | 0.1908319 | 0.6123309 |  | 0.7139198 | 11.95716 |  | -0.5230879 | 11.97283 | 0.965 |  | 0.5649131 |
| CO_2_LT+PDL VS PDL | _ | _ |  | _ | _ |  | _ | _ | _ |  | _ |
| ESWT+RT VS RT | 1.011314 | 0.465274 |  | 0.3948108 | 44.7448 |  | 0.6165029 | 44.7472 | 0.989 |  | 0.5645978 |
| MT VS RT | 1.923109 | 0.7029934 |  | 0.3288559 | 63.26319 |  | 1.594253 | 63.26717 | 0.980 |  | 0.5645401 |
| OPLT+RT VS RT | 1.034456 | 0.6879113 |  | 0.384156 | 63.26649 |  | 0.6503 | 63.27023 | 0.992 |  | 0.5645402 |
| PT VS PTS | -0.0032679 | 0.4865919 |  | 1.392196 | 44.76253 |  | -1.395464 | 44.76524 | 0.975 |  | 0.5646973 |
| PT VS RT | 0.890054 | 0.6838644 |  | 0.1539012 | 21.09666 |  | 0.7361529 | 21.10821 | 0.972 |  | 0.564751 |

**Table 2. The result of nodesplit in scar thickness.**

| side | Direct | |  | Indirect | |  | Difference | | |  | tau |
| --- | --- | --- | --- | --- | --- | --- | --- | --- | --- | --- | --- |
|  | coef. | Std.Err |  | coef. | Std.Err |  | coef. | Std.Err | *P* >∣Z∣ |  |  |
| CO_2_LT VS RT | 1.747142 | 4.4126145 |  | 0.3494839 | 12.91115 |  | 1.397658 | 12.91774 | 0.914 |  | 0.3038639 |
| ESWT+RT VS RT | 1.061926 | 0.2560795 |  | 3.492004 | 36.52202 |  | -2.430079 | 36.52292 | 0.947 |  | 0.3038286 |
| MT+RT VS RT | 0.0607113 | 0.3544715 |  | 3.492024 | 63.25668 |  | -3.431313 | 63.25767 | 0.957 |  | 0.3038104 |
| PT VS PTS | _ | _ |  | _ | _ |  | _ | _ | _ |  | _ |
| PT VS RT | 0.0793625 | 0.4796707 |  | 0.9103347 | 0.9410303 |  | -0.8309722 | 1.012602 | 0.412 |  | 0.3290169 |
| PT VS SG | _ | _ |  | _ | _ |  | _ | _ | _ |  | _ |
| PTS VS RT | 0.4978427 | 0.3558071 |  | -1.108015 | 0.5814412 |  | 1.605858 | 0.6187452 | 0.009 |  | 1.08e-10 |
| PTS VS SG | _ | _ |  | _ | _ |  | _ | _ | _ |  | _ |
| RT VS SG | -0.1929821 | 0.4939441 |  | -0.9684586 | 0.9526519 |  | 0.7754765 | 1.036775 | 0.454 |  | 0.3390451 |

**Table 3. The result of nodesplit in VAS.**

| side | Direct | |  | Indirect | |  | Difference | | |  | tau |
| --- | --- | --- | --- | --- | --- | --- | --- | --- | --- | --- | --- |
|  | coef. | Std.Err |  | coef. | Std.Err |  | coef. | Std.Err | *P* >∣Z∣ |  |  |
| ESWT+RT VS RT | 1.03856 | 3.297411 |  | -0.1541504 | 15.89675 |  | 1.19271 | 16.23513 | 0.941 |  | 3.282873 |
| HILT VS RT | 0.9049957 | 3.250927 |  | 2.010879 | 63.81632 |  | -1.105884 | 63.89904 | 0.986 |  | 3.236913 |
| MT+RT VS RT | -1.296006 | 2.310153 |  | 2.055744 | 45.36154 |  | -3.35175 | 45.42035 | 0.941 |  | 3.251321 |
| PT +RT VS PTS+RT | _ | _ |  | _ | _ |  | _ | _ | _ |  | _ |
| PT +RT VS RT | -1.260702 | 3.25565 |  | 1.382716 | 63.81642 |  | -1.508786 | 63.9001 | 0.981 |  | 3.236875 |
| PT +RT VS SG+RT | _ | _ |  | _ | _ |  | _ | _ | _ |  | _ |
| PTS+RT VS RT | 0.6471587 | 3.256545 |  | 2.155946 | 63.81662 |  | -1.508787 | 63.9003 | 0.981 |  | 3.236877 |
| PTS+RT VS SG+RT | _ | _ |  | _ | _ |  | _ | _ | _ |  | _ |
| RT VS SG+RT | -0.3740074 | 3.256856 |  | -1.882794 | 63.81692 |  | 1.508787 | 63.90057 | 0.981 |  | 3.236884 |

We acknowledge the importance of exploring the relative benefits and risks of different non-surgical treatments for post-burn scars. In future research, we plan to conduct additional sensitivity analyses and subgroup analyses to better understand the impact of various treatment modalities, including ablative CO_2_ laser and microneedling therapies under general anaesthetic. This will help clinicians make more informed decisions based on the specific needs and circumstances of their patients. Thank you again for your thoughtful comments.

Reviewer 2:

***Comment 1: Abstract:Much improved. Why is SMC only mentioned in presentation of some results eg associated with ECSWT? Isn’t SMC implied in most studies as the control condition for all treatments included regardless of whether there is a stated comparison with routine care? Please drop it and be consistent with terminology in Abstract as well as the remainder of the manuscript. Consider that SMT differs between studies and is not necessarily the same for each study where that condition is stated. Perhaps ‘routine local treatment’ or similar is a better generic terminology?* Response**: Thank you for this important and thoughtful comment. We agree with you that the term SMC may imply a uniform control condition across all studies, which is not always accurate, as the actual content of standard care can vary between centers and studies. To improve consistency and clarity in terminology throughout the manuscript, we have revised the text to use the more general and descriptive term "routine treatment" instead of SMC.

***Comment 2: Introduction:Much improved. Thank you.* Response**: Thank you very much for your kind comment regarding the revised Introduction section. We carefully revised this part based on your previous suggestions. We are pleased that the changes have been recognized and appreciate your constructive support.

***Comment 3: Methods:Please confirm that CO2LT only refers to the ablative procedure, and does not include pooled results from the non-ablative intervention (which is referred to in Discussion).* Response**: Thank you for this important clarification. In this study, We confirm that CO₂LT refers exclusively to the ablative CO₂LT procedure, and does not include any pooled results from non-ablative CO₂LT interventions. Although non-ablative approaches are mentioned in the Discussion section for contextual and illustrative purposes, they were not included in the data extraction, treatment classification, or network meta-analysis. We have revised the Methods section accordingly to explicitly clarify this point and enhance transparency. These changes are on pages 7-8 of the revised manuscript, as shown below:

Articles were included if (1) they were published in peer-reviewed journals; (2) they reported non-surgical treatments for patients with post-burn scars, including monotherapies or combination therapies; (3) they reported the following outcomes at approximately 6 months post-injury: VSS score, scar thickness measured by ultrasound, and pain intensity assessed via VAS score; (4) they were randomized controlled trials (RCTs). In this NMA, CO₂LT refers specifically to the ablative procedure.

***Comment 4: Methods: Please add a short summary of the response points to justify conference abstract exclusion in Section 2.3.* Response**: Thank you for this helpful suggestion. According to your suggestions, we have added a brief justification for the exclusion of conference abstracts in Section 2.3 of the revised manuscript. Specifically, we clarify that conference abstracts were excluded due to concerns regarding incomplete reporting of study methods, intervention details, and outcome data, which could limit the accuracy of risk of bias assessment and increase the potential for selective outcome reporting. These changes are on page 8 of the revised manuscript, as shown below:

Articles were excluded if (1) they were clinical trials, review articles, letters to the editor or case series; (2) they were not published in English; (3) they reported incomplete data; (4) the full text was unavailable. Additionally, conference abstracts were excluded due to insufficient detail on study design, interventions, and outcomes, which would limit the ability to accurately assess risk of bias and interpret treatment effects.

***Comment 5: Results: Well done. Section 3.3.1, sentence 3 - ….therefore the consistency model was used for the NMA….’ Please change ‘was used’ to ‘was applicable’ or similar.* Response**: Thanks for your valuable comments. We agree with your recommendation. We have revised the sentence from “…therefore the consistency model was used for the NMA…” to “…therefore the consistency model was applicable for the NMA…”in Section 3.3.1, Section 3.3.2, and Section 3.3.3.

***Comment 6: Results: Section 3.2 and Discussion (Strengths and Limitations) still refers to Table 1, 3.31 refers to Table 2, Section 3.3.2 refers to Table 3, Section 3.3.3 refers to Table 4 – all of which have not been included in the revised submission. Please remove reference to all tables from the text if they are not going to be part of the manuscript.* Response**: Thank you for bringing this to our attention. We apologize for any confusion and confirm that all tables (Table 1–4) have been included in the revised manuscript submission. It is possible that these files were not properly attached or viewed during the previous upload. We have carefully ensured that all relevant tables are now uploaded as part of the manuscript and referenced appropriately in the text (Section 3.2, 3.3.1, 3.3.2, and 3.3.3).

***Comment 7: Discussion: With respect to Limitations - Please consider and comment on whether the choice of timeframe for the primary study outcome impacts the comparison of effect size. For instance, how many burn services apply ablative CO2LT prior to 6 months post-burn? Does the study (review) method therefore bias or impact the volume of studies that support the effect size of various interventions?* Response**: Thank you for raising this important methodological consideration regarding the impact of our selected outcome timeframe on the interpretation of treatment effects. We acknowledge that the choice of a 6-month post-injury timepoint as the primary endpoint may influence the comparability of effect sizes across interventions. Scar maturation is a dynamic process, and different treatments may be applied at varying stages during recovery depending on local clinical practices and patient-specific factors.

As you correctly notes, some burn centers may initiate ablative CO₂LT earlier than 6 months post-injury, while others may delay intervention until later in the healing process. By selecting 6 months as the common timepoint for outcome assessment, we aimed to capture scar characteristics at a clinically relevant phase of maturation while ensuring sufficient data availability across studies. However, we agree that this approach may have excluded studies with earlier or more prolonged follow-up periods, potentially affecting the volume of evidence available for certain interventions and introducing selection bias.

In the revised manuscript, we have added a paragraph in the Limitations section to explicitly address this concern:

This NMA focuses on outcomes assessed at approximately 6 months post-injury. This timepoint may not reflect the optimal timing for all interventions (such as CO₂LT), and it may limit the number of eligible studies reporting outcomes within this window. As a result, the estimated effect sizes may not fully represent the potential benefits of interventions delivered earlier or later in the recovery trajectory.

***Comment 8: Discussion: Consider specifically identifying the gaps in the research literature, framed by the Global Top 10 Research Priorities (Richards et al, Lancet Global Health, 2025) and directions for future research based on the review findings.* Response**: Thank you for your valuable suggestion. We fully agree with you that situating our findings within the context of the Global Top 10 Research Priorities for Burn Care (Richards et al., The Lancet Global Health, 2025) enhances the relevance and applicability of our review. In response, we have expanded the Discussion section to explicitly identify key research gaps in the current literature on non-surgical interventions for post-burn scars, and to propose future research directions aligned with these global priorities. The revised discussion is shown below:

In line with the Global Top 10 Research Priorities for Burn Care, this NMA identified several critical gaps in the current evidence base: (1) a lack of long-term follow-up data on the efficacy and safety of non-surgical treatments; (2) underutilization of patient-reported outcome measures (PROMs), such as POSAS, which limits understanding of patient-centered impacts beyond clinician-reported metrics like VSS or VAS; (3) limited reporting of cost-effectiveness or resource implications, which hinders informed decision-making in low- and middle-income settings; (4) insufficient standardization of outcome reporting across trials, contributing to heterogeneity and limiting comparability.

Future research should prioritize addressing these gaps to generate more robust, equitable, and clinically meaningful evidence in post-burn scar management. Specifically, we recommend conducting economic evaluations of promising interventions—particularly in resource-limited settings—and developing core outcome sets for post-burn scar interventions to support evidence synthesis and guideline development.

***Comment 9: Conclusions: Remove the reference to SMT as it is confusing.* Response**: Thank you for your comment. We would like to clarify that the term SMT does not appear in the current version of the Conclusions section. In earlier versions of the manuscript, we used the term standard medical care (SMC) to refer to the control condition; however, following previous your feedback, we have now replaced this with the more general and descriptive term "routine treatment" throughout the manuscript — including in the Conclusions — to avoid confusion and reflect the variability in standard care across studies. The revised conclusions is shown as below：

The result of this NMA illustrates that MT, CO_2_LT, ESWT combined with RT are the most effective non-surgical treatments for reducing VSS score, scar thickness and VAS score, respectively. However, it is important to note that this conclusion is drawn from a limited number of studies. And the findings reflect outcomes at a specific stage of scar maturation (approximately 6 months post-burn). It is advocated for future trials to adopt validated multidimensional tools like POSAS to enhance consistency and reliability in scar evaluation. Additionally, well-designed randomized controlled trials with a large sample size are needed to validate these findings in the future.

***Comment 10: Figures: Remove SMC from ALL figures please, except where it is itself the defined group or comparison.* Response:** Thanks for your comment. Regarding your request to remove SMC from all figures, we understand your concern about clarity and the presentation of the data. However, in our network meta-analysis, SMC (according to comment 1, SMC was revised RT) serves as the control intervention and is a critical component for comparison across different studies. This is particularly important in the context of our analysis, where RT provides a baseline for evaluating the effectiveness of other interventions.

Specifically, in the network plot, RT is essential to illustrate the connections and comparisons between different interventions. Removing RT would disrupt the integrity of the network and could potentially mislead readers about the relationships and comparisons being made.

Similarly, in the cumulative ranking curve and the surface under the cumulative ranking curve plot, RT is included to provide a comprehensive view of the relative effectiveness of all interventions, including the control. These figures are designed to show the full spectrum of treatment effects, and excluding RT would omit a significant part of the analysis.

For the funnel plot, RT is included to assess publication bias across all studies, including those comparing interventions to the standard care. Removing RT from this plot would compromise the completeness of the bias assessment.

We have carefully reviewed all figures and believe that retaining RT is necessary for the accuracy and completeness of our analysis. We are committed to ensuring that our figures are as clear and informative as possible. We hope this explanation clarifies the importance of including RT in our figures.

***Comment 11: Figures: How is the reader supposed to interpret Figures 3B, 4B, 5B. Footnotes or explanations in captions are required to help the reader.***

**Response**: Thank you for your valuable feedback regarding Figures 3B, 4B, 5B. We agree that additional explanations are necessary to help readers understand the cumulative probability plots presented in Figure 3B, 4B, 5B. To address this, we have revised the Figures as follows:

**Fig 3B.** Plot of the surface under the cumulative ranking curve for VSS score.

CO_2_LT: CO_2_ Laser Therapy (the SUCRA value is 28.6%), CO_2_LT+PDL: CO_2_ Laser Therapy+Pulsed dye laser, ESWT+RT (the SUCRA value is 63.5%): Extracorporeal Shock Wave Therapy+Routine Treatment (the SUCRA value is 63.5%), MT: Massage Therapy (the SUCRA value is 89.0%), OPLT+RT: Orange Polarized Light Therapy+Routine Treatment (the SUCRA value is 63.0%), PDL: Pulsed dye laser (the SUCRA value is 23.5%), PT: Pressure Therapy (the SUCRA value is 57.9%), PTS: Pressure Garments and Silicone (the SUCRA value is 57.3%), RT : Routine Treatment (the SUCRA value is 18.8%). The overall position of the curve corresponds with the SUCRA value reported in the results. A higher curve indicates a greater likelihood of being ranked higher.

**Fig 4B.** Plot of the surface under the cumulative ranking curve for scar thickness.

CO_2_LT: CO_2_ Laser Therapy (the SUCRA value is 96.8%), ESWT+RT: Extracorporeal Shock Wave Therapy+Routine Treatment (the SUCRA value is 82.0%), MT+RT: Massage Therapy+Routine Treatment (the SUCRA value is 28.6%), PT: Pressure Therapy (the SUCRA value is 37.6%), PTS: Pressure Garments and Silicone (the SUCRA value is 35.4%), SG: Silicone Gel (the SUCRA value is 47.5%), RT: Routine Treatment (the SUCRA value is 22.1%). The overall position of the curve corresponds with the SUCRA value reported in the results. A higher curve indicates a greater likelihood of being ranked higher.

**Fig 5B.** Plot of the surface under the cumulative ranking curve for VAS score.

ESWT+RT: Extracorporeal Shock Wave Therapy+Routine Treatment (the SUCRA value is 58.6%), HILT: High Intensity Laser Therapy (the SUCRA value is 57.6%), MT+RT: Massage Therapy+Routine Treatment (the SUCRA value is 31.6%), PT+RT: Pressure Therapy+Routine Treatment (the SUCRA value is 46.2%), PTS+RT: Pressure Garments and Silicone+Routine Treatment (the SUCRA value is 56.1%), SG+RT: Silicone Gel+Routine Treatment (the SUCRA value is 52.0%), RT: Routine Treatment (the SUCRA value is 47.9%). The overall position of the curve corresponds with the SUCRA value reported in the results. A higher curve indicates a greater likelihood of being ranked higher.

We believe these changes will significantly enhance the reader's understanding of Figures 3B, 4B, 5B.
