# Supplementary material for: Non-surgical treatments for post-burn scars: A network meta-analysis
Source: PLoS One. 2025 Aug 21;20(8):e0330428. doi: 10.1371/journal.pone.0330428 (PMC12370048; doi:10.1371/journal.pone.0330428)
Supplement: Attachment — Submitted filename: Response_to_Reviewers_auresp_3.docx [file pone.0330428.s007.docx]

Dear Editors and Reviewers,

Thank you very much for giving us opportunities to revise our manuscript, and we appreciate the reviewer a lot for his positive and constructive comments and suggestions. We have studied reviewer’s comments carefully and have made revisions. We hope the corrections will meet with your approval.

Journal Requirement 1:

***If the reviewer comments include a recommendation to cite specific previously published works, please review and evaluate these publications to determine whether they are relevant and should be cited. There is no requirement to cite these works unless the editor has indicated otherwise. Please review your reference list to ensure that it is complete and correct. If you have cited papers that have been retracted, please include the rationale for doing so in the manuscript text, or remove these references and replace them with relevant current references. Any changes to the reference list should be mentioned in the rebuttal letter that accompanies your revised manuscript. If you need to cite a retracted article, indicate the article’s retracted status in the References list and also include a citation and full reference for the retraction notice.***

**Response**: Thank you for this helpful guidance. We have carefully reviewed all references cited in our manuscript and confirm the following:

(1) No retracted articles are included in the reference list.
(2) All references are complete, accurate, and formatted in accordance with the journal’s instructions.
(3) No previously suggested works have been omitted. Any additional studies recommended by the reviewers were evaluated for relevance and incorporated only when they met our inclusion criteria.

Thank you again for your thoughtful comments.

Reviewer 2:

***Comment 1: Please adjust this added sentence to Limitations to include ‘contemporary’: In addition, one limitation of this NMA is the exclusion of studies reporting only POSAS outcomes, which may have led to the omission of more recent, CONTEMPORARY interventions that adopt this more comprehensive and patient-centered assessment tool.***

**Response**: Thank you for your valuable suggestion. We have revised the limitation section as recommended to include the term “contemporary”. The revised sentence is shown as below:

“In addition, one limitation of this NMA is the exclusion of studies reporting only POSAS outcomes, which may have led to the omission of more recent, contemporary interventions that adopt this more comprehensive and patient-centered assessment tool.”

***Comment 2: Unfortunately, the additional tables provided are unformatted and uninterpretable. It is fair that the authors are avoiding a sensitivity analysis which removes combined results from (ablative) CO2 laser and microneedling under general anaesthetic studies due to statistical power. However, in reply (2) (clinical heterogeneity), the authors contradict themselves and confirm the fundamental differences between the CO2 laser and needling sub-set of techniques in comparison with all others, basically justifying the request for a sensitivity analysis. However, so as not to create an impasse, if the authors choose not to include a sensitivity analysis (perhaps only removing CO2 laser studies), please instead add a statement in Discussion or Limitations which reiterates to the reader the reasons for applying the definition of non-surgical techniques in this review.***

**Response**: Thank you for your detailed and constructive feedback. We sincerely apologize for the formatting issues in the supplementary tables submitted in our previous revision. We fully acknowledge the reviewer’s concern regarding their readability and interpretability. In response, we have now carefully reformatted all supplementary tables to ensure clarity, consistency, and alignment with standard academic presentation practices. The revised tables are provided in the updated submission.

Concerning the sensitivity analysis, we fully acknowledge the reviewer’s point regarding the apparent contradiction between our justification for not conducting a sensitivity analysis (due to limited statistical power) and our recognition of the clinical heterogeneity among non-surgical techniques, particularly between CO₂ laser/microneedling under general anaesthesia and other less invasive modalities. We agree that this heterogeneity indeed supports the rationale for a sensitivity analysis.

However, in light of the limited number of studies available for comparison and to avoid compromising the interpretability of the results, we have opted not to perform an additional sensitivity analysis excluding these studies. As suggested by the reviewer, we have added a clear statement in the Limitations section (page 24), explaining the rationale behind our working definition of “non-surgical treatments”, as shown below:

It should be noted that all interventions not involving excisional surgery were classified as “non-surgical” in this review. This definition intentionally includes ablative CO₂ laser and fully ablative microneedling procedures, even when performed under general anaesthesia. Though these modalities differ from other non-surgical interventions in terms of injury depth, perioperative requirements, and expected morbidity, a consistent classification was adopted to enable a comprehensive synthesis of the available evidence on non-surgical approaches for post-burn scar management. However, this approach inevitably introduces clinical heterogeneity across the included studies, which may affect the precision and generalizability of the pooled estimates. Readers are therefore advised to interpret the findings with due consideration of the inherent differences among the non-surgical interventions included.

***Comment 3 (Methods): Data Extraction, page 8 – Please change adjective to ‘closest’ if this is an accurate description to: For outcomes reported at multiple time points, only data CLOSEST to 6 months were extracted.* Response**: Thanks for your constructive suggestion. We agree that the term “closest” more accurately conveys the intended meaning. Accordingly, we have revised the sentence on page 8 as follows:

“For outcomes reported at multiple time points, only data closest to 6 months were extracted.”

***Comment 4 (Figures): Please note, the previous comment was not a request to remove SMC from the figures, it was a reference to remove the title which was confusing. In that, SMC has now been changed to RT appropriately, thank you.***

**Response**: Thank you for the clarification. We apologize for the misunderstanding in our previous revision. As per your suggestion, we have removed the confusing title (previously labeled as “SMC”) from the figures and have retained “RT” as the appropriate label throughout the manuscript. We appreciate your guidance on this matter.

***Comment 5 (Figures): Please adjust the last sentence of the footnotes added in the last version: A higher curve indicates a greater likelihood of being ranked higher. This explanation is confusing and needs additional clarification and tweak of expression which removes duplication of the word ‘higher’.***

**Response**: Thank you for your helpful feedback. We agree that the original wording was repetitive and potentially confusing. Accordingly, we have revised the last sentence of the footnote to improve clarity and avoid duplication of the word “higher”. The revised sentence is shown as follows:

“A higher curve indicates better performance in terms of treatment ranking.”
