# Supplementary material for: Non-surgical treatments for post-burn scars: A network meta-analysis
Source: PLoS One. 2025 Aug 21;20(8):e0330428. doi: 10.1371/journal.pone.0330428 (PMC12370048; doi:10.1371/journal.pone.0330428)
Supplement: Attachment — Submitted filename: Response_to_Reviewers_auresp_4.docx [file pone.0330428.s008.docx]

Dear Editors and Reviewers,

Thank you very much for giving us opportunities to revise our manuscript, and we appreciate the reviewer a lot for his positive and constructive comments and suggestions. We have studied reviewer’s comments carefully and have made revisions. We hope the corrections will meet with your approval.

Journal Requirement 1:

***If the reviewer comments include a recommendation to cite specific previously published works, please review and evaluate these publications to determine whether they are relevant and should be cited. There is no requirement to cite these works unless the editor has indicated otherwise. Please review your reference list to ensure that it is complete and correct. If you have cited papers that have been retracted, please include the rationale for doing so in the manuscript text, or remove these references and replace them with relevant current references. Any changes to the reference list should be mentioned in the rebuttal letter that accompanies your revised manuscript. If you need to cite a retracted article, indicate the article’s retracted status in the References list and also include a citation and full reference for the retraction notice.***

**Response**: We would like to thank the journal for the guidelines regarding the reference list. In response to the instructions, we have carefully reviewed all references cited in our manuscript to ensure completeness, accuracy, and relevance. We evaluated any references suggested by the reviewers and have included those that are pertinent to our study in the revised manuscript. Additionally, we have verified that none of the cited works have been retracted. All references are up-to-date, relevant, and appropriately formatted according to the journal’s citation style.

Thank you again for this thoughtful comments.

Editors :

***Comment 1: We note that the grant information you provided in the “Funding Information” and “Financial Disclosure” sections do not match. When you resubmit, please ensure that you provide the correct grant numbers for the awards you received for your study in the “Funding Information” section.***

**Response**: We sincerely thank the editor for the comments and for handling our manuscript. We have updated the “Funding Information” section to ensure that it contains the correct grant numbers, as shown below:

Find a Funder: Sichuan Province Science and Technology Program.

Award Number: 23ZDYF1907.

Grant Recipient: Not applicable.

***Comment 2: Please note that funding information should not appear in the Acknowledgments section or other areas of your manuscript. We will only publish funding information present in the Funding Statement section of the online submission form. Please remove any funding-related text from the manuscript.***

**Response**: Thanks for your helpful comment. We have carefully removed all funding-related information from the Acknowledgments section and any other parts of the manuscript, as requested. The funding details are now only provided in the Funding Information section of the online submission form, in accordance with the journal’s policy.

Reviewer 2:

***Comment 1: Define (or remove) the acronym SUCRA.***

**Response**: Thank you for your valuable comment. As suggested, we have added the full term “surface under the cumulative ranking curve” at its first mention in the Abstract section, as shown below:

A total of 17 studies and 1,013 participants were included in this analysis. The treatment ranking revealed that massage therapy demonstrated the most significant efficacy in reducing Vancouver Scar Scale score (surface under the cumulative ranking curve [SUCRA] = 89.0%), CO_2_ laser therapy exhibited the highest efficacy in decreasing scar thickness (SUCRA=96.8%) , and extracorporeal shock wave therapy + routine treatment showed the most significant efficacy in reducing Visual Analogue Scale score (SUCRA=58.6%).

***Comment 2: To rebalance the word count – remove sentence about risk of bias, it is not necessary.***

**Response**: Thanks for your valuable comment. As suggested, we have removed the sentence regarding the risk of bias assessment to rebalance the word count. The revised abstract now is shown as follows:

PubMed, Web of Science, Cochrane Library, PEDro, and Embase were systematically searched for eligible randomized controlled trial studies, and the network meta-analysis was performed via a frequentist approach. The primary outcomes assessed were Vancouver Scar Scale score, scar thickness and Visual Analogue Scale score.
